# Nutrient utilization and degradative enzyme activity of the dragon fruit canker pathogen, Neoscytalidium dimidiatum

**DOI:** 10.1099/mic.0.001727

**Published:** 2026-06-16

**Authors:** Rachel E. Kalicharan, Romina Gazis, Jessie Fernandez

**Affiliations:** 1 Department of Microbiology and Cell Science, University of Florida, Gainesville, Florida, 32611, USA; 2 Department of Plant Pathology, Tropical Research and Education Center, University of Florida, Homestead, Florida, 33031, USA

**Keywords:** dragon fruit canker, *Neoscytalidium dimidiatum*, nutrient utilization, PCWDE, CAZymes

## Abstract

Dragon fruit canker (DFC), caused by *Neoscytalidium dimidiatum*, is a major disease affecting dragon fruit production. However, the pathogen’s nutrient utilization strategies and enzymatic activities remain poorly characterized. Using calcofluor white-based fluorescence microscopy, we show that nutrient limitation restricts fungal growth and development, impairing melanization and arthrosporulation. Carbon utilization assays revealed a preference for maltose, suggesting adaptation to starch-derived sugars. Nitrogen utilization assays indicated efficient assimilation of complex organic nitrogen sources. In addition, we experimentally validated extracellular enzymatic activities involved in host macromolecule degradation, including cellulase, amylase, pectinase and protease activities. Together, these findings provide insight into the physiological traits of *N. dimidiatum* associated with growth and development under varying nutrient conditions.

## Introduction

 Dragon fruit (*Selenicereus spp*., syn. *Hylocereus spp*.)*,* commonly known as pitaya, is an important perennial tropical crop cultivated worldwide [1]. With rising global demands, the dragon fruit industry has grown into a billion-dollar market, producing millions of metric tons of fruit annually [2]. Despite this rapid expansion, production is increasingly jeopardized by dragon fruit canker (DFC), caused by the fungal pathogen *Neoscytalidium dimidiatum*. This pathogen is notorious for its broad host range, causing dermatological infections in humans and diseases in woody and herbaceous plants, including dragon fruit [3,8].

DFC is considered the most destructive disease of dragon fruit globally, with severe economic losses reaching up to 80% [5]. Infection occurs in vegetative tissues and fruit through natural openings, such as stomata or wounds and can also involve direct penetration via appressoria [9]. Symptoms appear as sunken, chlorotic spots on cladodes that progress to necrotic lesions associated with pycnidia formation and conidia production, ultimately leading to characteristic ‘shot-hole’ symptoms [10,11].

Given its aggressive nature, most research has focused on practical management strategies, including early detection, fungicide application and sanitation practices [5,10, 12]. Recently, molecular studies have begun to elucidate the basis of *N. dimidiatum* pathogenicity, including whole-genome sequencing, effector identification, host defence gene expression, metabolite profiling and biocontrol exploration [9,13, 14]. However, critical aspects, such as nutrient preference/utilization and validation of host-degradative enzymes, remain poorly understood. Here, we investigate carbon and nitrogen utilization preferences in *N. dimidiatum* and evaluate extracellular enzyme activity associated with plant macromolecule degradation. We also examine the impact of nutrient availability on fungal development, melanization and arthrosporulation. This work provides insight into the physiological traits of *N. dimidiatum* relevant to nutrient acquisition and fungal development.

## Methods

### Fungal strains

The fungal strain *N. dimidiatum* was provided by Dr. Romina Gazis (University of Florida). Cultures were maintained on potato dextrose agar (PDA) and incubated at 28 °C in the dark.

### Carbon and nitrogen source testing

Carbon and nitrogen preferences were assessed by transferring 4 mm agar plugs from 7-day-old PDA cultures onto modified Czapek-Dox minimal medium (MM; 1 g K_₂_HPO_₄_, 0.5 g MgSO_₄_·7 H_₂_O, 0.5 g KCl, 0.01 g FeSO_₄_·7H_₂_O, pH 6.5, solidified with 15 g/L agar). For carbon utilization assays, carbon-free minimal medium (CFMM) was supplemented with 1% (wt/vol.) of each individual carbon source. For nitrogen utilization assays, nitrogen-free minimal medium (NFMM) was supplemented with 0.1% (wt/vol.) of each nitrogen source. Plates were incubated at 28 °C, and colony morphology, pigmentation and arthrospore production were evaluated at 3, 5 and 7 days post-inoculation (dpi).

### Arthrosporulation assay

Arthrospores were harvested from cultures at 3, 5 or 7 dpi and filtered through three layers of Miracloth. Suspensions were centrifuged at 3,900 r.p.m. for 5 min, the supernatant was discarded and pellets were resuspended in 1 mL of water. Arthrospore counts were determined in triplicate using a hemocytometer.

### Heat map generation and scoring criteria for melanization and arthrosporulation

**Fig. 1.**
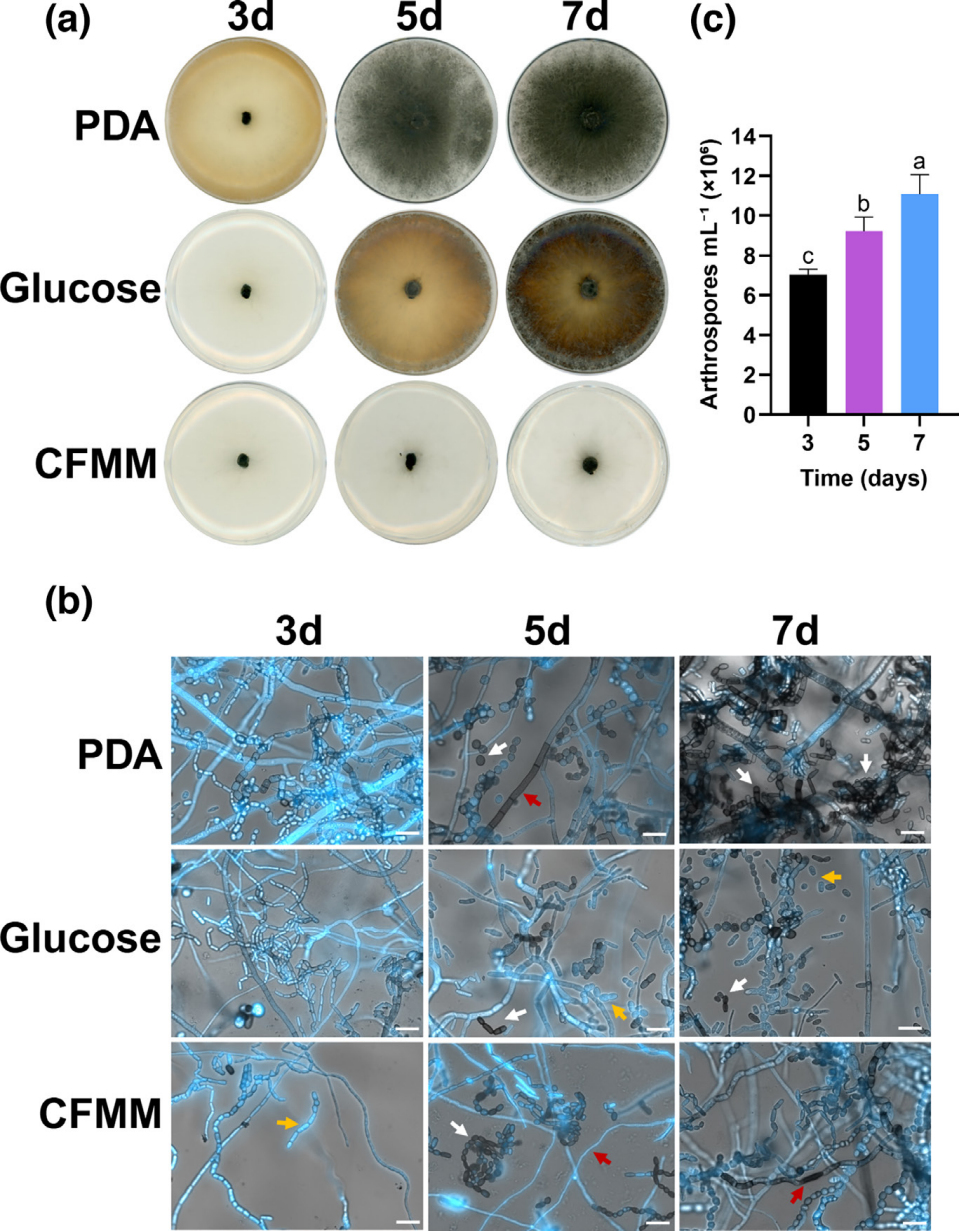
Development and melanization of *N. dimidiatum* under various nutrient conditions. (**a)** Colony morphology of *N. dimidiatum* grown on potato dextrose agar (PDA), carbon-free minimal medium (CFMM) or CFMM supplemented with 1% (wt/vol.) glucose at 3, 5 and 7 dpi. (**b)** Calcofluor white fluorescence microscopy of cultures grown under the indicated conditions at 3, 5 and 7 dpi. White arrows indicate melanized arthrospores, orange arrows indicate non-melanized spores, and red arrows indicate hyphae. Scale bar=20 µm. (**c)** Arthrospore concentration of *N. dimidiatum* grown on PDA at 3, 5 and 7 dpi. Values represent mean±sd (*n*=3). One-way ANOVA with Tukey’s multiple comparisons test; *P* <0.05.

Melanization and arthrosporulation were scored on a four-point scale (0–3) and visualized as heatmaps. Melanization scores were defined based on the reference phenotypes shown in Fig. 1 : 0, minimal colony growth with no/trace melanization; 1, full plate coverage with little melanization; 2, full coverage with moderate melanization; and 3, full coverage with strong melanization. Arthrosporulation scores were assigned based on arthrospore concentration ranges: 0, ≤5×10^4^ arthrospores/mL; 1, 5.01×10^4^–5×10^5^ arthrospores/mL; 2, 5.01×10^5^–5×10^6^ arthrospores/mL; and 3, ≥5.01×10^6^ arthrospores/mL. Scores were mapped to the corresponding colour for visualization.

### Qualitative enzyme activity assays

4 mm agar plugs from 7-day-old *N. dimidiatum* cultures were placed on MM supplemented with 1% wt/vol. carboxymethylcellulose (CMC) sodium salt (TCI), 1% wt/vol. starch, 1% wt/vol. citrus pectin (Sigma), or 10% wt/vol. skim milk (SM). Plates were incubated at 28 °C for 3 days before evaluation. For cellulase activity, cellulose-supplemented plates were stained with 0.1% Congo Red solution for 15 min, cleared with three 5 min washes in 1 M NaCl, and the zones of clearance were assessed. For amylase and pectinase activity, starch and pectin-supplemented plates were stained with Gram’s iodine for 5 min (amylase) or 10 min (pectinase), excess iodine was poured off, and starch/pectin degradation was assessed by staining intensity and the presence of a zone of clearance. For protease activity, the zone of clearance was assessed on SM-supplemented plates.

### Fungal cell microscopy

Microscopic analysis of fungal development was performed on PDA, CFMM and CFMM supplemented with 1% (wt/vol.) glucose. Medium was applied to autoclaved microscope slides and 10 µL of a spore suspension (1×10^4^ arthrospores/mL) was inoculated. Slides were incubated at 28 °C in humid chambers and imaged at 3, 5 and 7 dpi using a Zeiss Axio Observer 7 microscope. For calcofluor white (CFW) staining, samples were treated with one drop of CFW (Sigma) and one drop of 10% KOH per 3 cm of slide, incubated for 1 min and imaged. Images were processed using Zen Microscopy Software v3.13 (Zeiss).

### Statistical analysis

Statistical analyses were performed using a one-way ANOVA and a two-way ANOVA, with Dunnett post-hoc and Tukey post-hoc tests, comparing each treatment to the control. All experiments included at least three biological replicates, each with three replicates. Differences were considered statistically significant at *P*<0.05.

## Results

### Nutrient deficiency impacts melanization and arthrosporulation of *N. dimidiatum*

To assess how nutrient availability influences the growth, development and maturation (arthrosporulation and melanization) of *N. dimidiatum*, cultures were established on PDA, CFMM and CFMM supplemented with 1% glucose. At 3 dpi, colonies on PDA exhibited extensive mycelial expansion but remained white, indicating early developmental stages. Similar vegetative growth was observed on CFMM and glucose-supplemented CFMM, with all cultures appearing predominantly white (Fig. 1a). Samples were stained with CFW, which binds to chitin and β-glucans in the fungal cell wall and fluoresces upon binding (Fig. 1b) [15]. Fluorescence intensity was comparable across all conditions, consistent with the presence of non-melanized hyphae and arthrospores.

By 5 dpi, clear differences in maturation emerged and were largely complete by 7 dpi under nutrient-rich conditions (Fig. 1a). PDA-grown cultures displayed pronounced melanization in both hyphae and arthrospores, whereas CFMM-grown cultures exhibited melanization primarily in arthrospores, with hyphae remaining largely non-melanized. Glucose-supplemented CFMM supported growth but showed minimal melanization in either hyphae or arthrospores (Fig. 1b). Correspondingly, PDA-grown samples and melanized arthrospores in CFMM exhibited reduced CFW fluorescence compared to glucose-grown samples, reflecting decreased dye accessibility in melanized cell walls [15]. Arthrosporulation was also reduced under CFMM and glucose conditions compared to PDA (Fig. 1b).

At 7 dpi, these developmental differences became more pronounced. PDA-grown cultures were fully melanized, forming dense hyphal networks and abundant arthrospores. In contrast, CFMM and glucose-grown cultures remained similar to the 5 dpi state, with melanization restricted to arthrospores but not hyphae, stronger overall CFW fluorescence and reduced arthrosporulation compared to PDA (Fig. 1b). Taken together, these observations indicate that nutrient limitation significantly impairs *N. dimidiatum* maturation, particularly affecting melanization and arthrosporulation.

### *N. dimidiatum* exhibits carbon-source preferences for maltose and glucose

To establish a baseline for development under nutrient-rich conditions, *N. dimidiatum* was cultured on PDA, where it exhibited robust growth, progressive melanization and abundant arthrosporulation (Fig. 1c). This phenotype served as a reference for evaluating the effects of nutrient limitation and defined carbon sources on fungal development.

Following our initial assessment, we evaluated the effect of individual carbon sources on fungal development. *N. dimidiatum* was grown on CFMM alone and CFMM supplemented with various carbon sources, including monosaccharides (glucose and galactose), polyols (glycerol, mannitol and sorbitol), disaccharides (sucrose, maltose and lactose) and the polysaccharide starch.

**Fig. 2.**
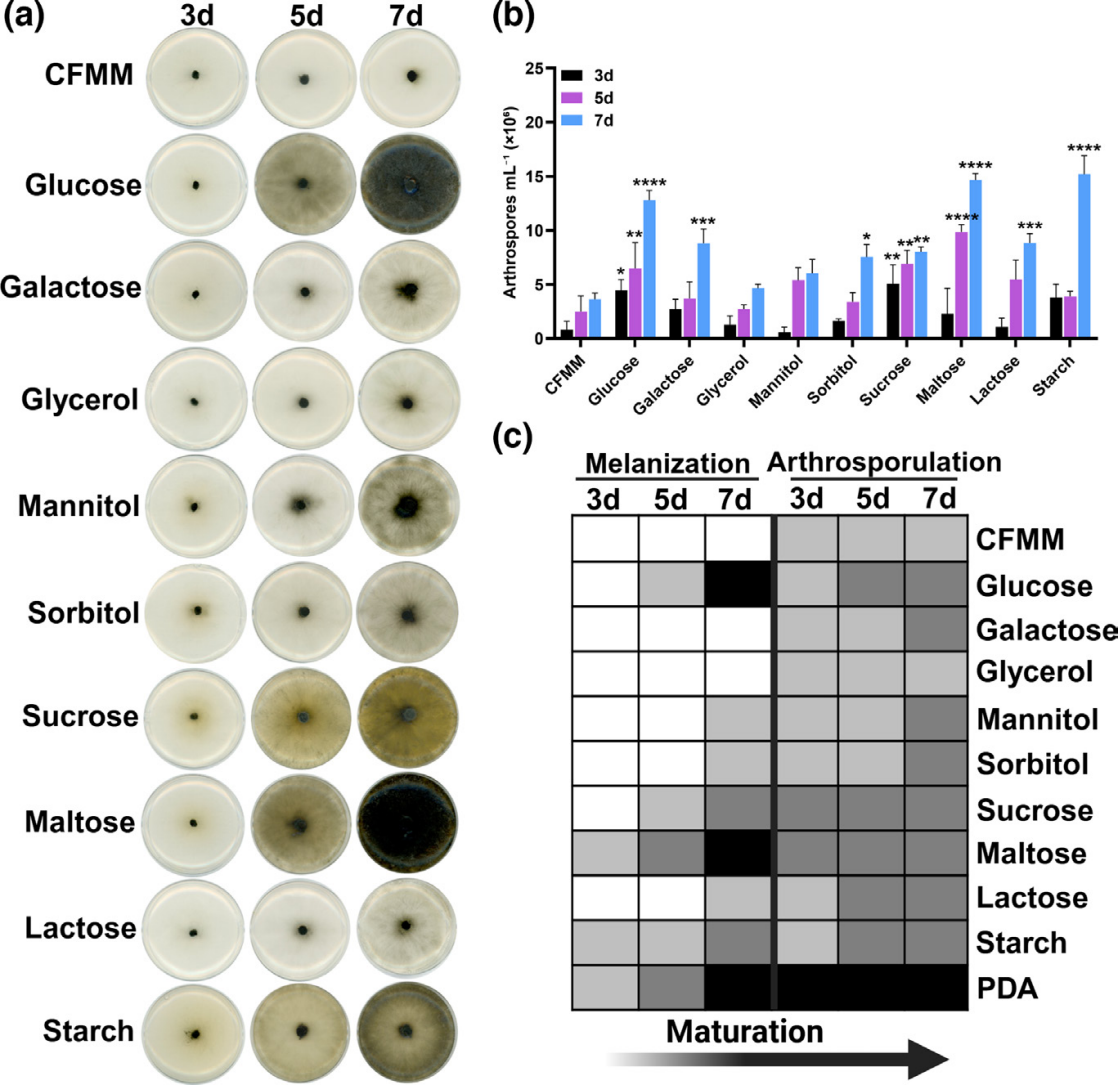
Carbon source utilization by *N. dimidiatum*. (a) Representative colony morphology of *N. dimidiatum* grown on carbon-free minimal medium (CFMM) supplemented with 1% (wt/vol.) individual carbon sources at 3, 5 and 7 dpi. **(b)** Quantification of arthrospore production under the indicated carbon sources. Statistical significance was determined by two-way ANOVA with Dunnett’s multiple comparisons test. * indicates *P* <0.05, ** indicates *P* <0.01, *** indicates *P*<0.001, **** indicates *P*<0.0001. **(c)** Composite heat map summarizing melanization and arthrosporulation scores across carbon sources. Darker shading indicates higher scores.

At 3 dpi, all treatments showed similar early-stage growth with no visible melanization (Fig. 2a). By 5 dpi, clear differences arose: glucose, sucrose and maltose supported faster colony expansion and earlier melanization. At 7 dpi, glucose- and maltose-supplemented cultures displayed the most advanced development, with extensive melanization and dense colonies.

Arthrospore quantification revealed that all carbon sources supported growth, but arthrosporulation varied significantly among treatments. Most supplemented media produced higher conidia counts than CFMM alone, with several carbon sources showing significantly increased arthrosporulation (*P*<0.05; Fig. 2b). A composite heat map (Fig. 2c) highlighted maltose and glucose as the most favourable carbon sources, promoting robust colony development, strong pigmentation and high arthrospore production. Other carbon sources, such as sucrose and starch, supported either melanization or arthrosporulation, but not both consistently. Notably, glycerol enhanced arthrosporulation relative to CFMM but yielded weak melanization and poor overall growth. Overall, these findings indicate that maltose and, to a lesser extent, glucose best support *N. dimidiatum* growth and maturation.

### *N. dimidiatum* demonstrates high affinity for complex, peptide-rich nitrogen sources

In addition to carbon preference, we assessed nitrogen utilization using NFMM alone or supplemented with diverse inorganic and organic nitrogen sources.

**Fig. 3.**
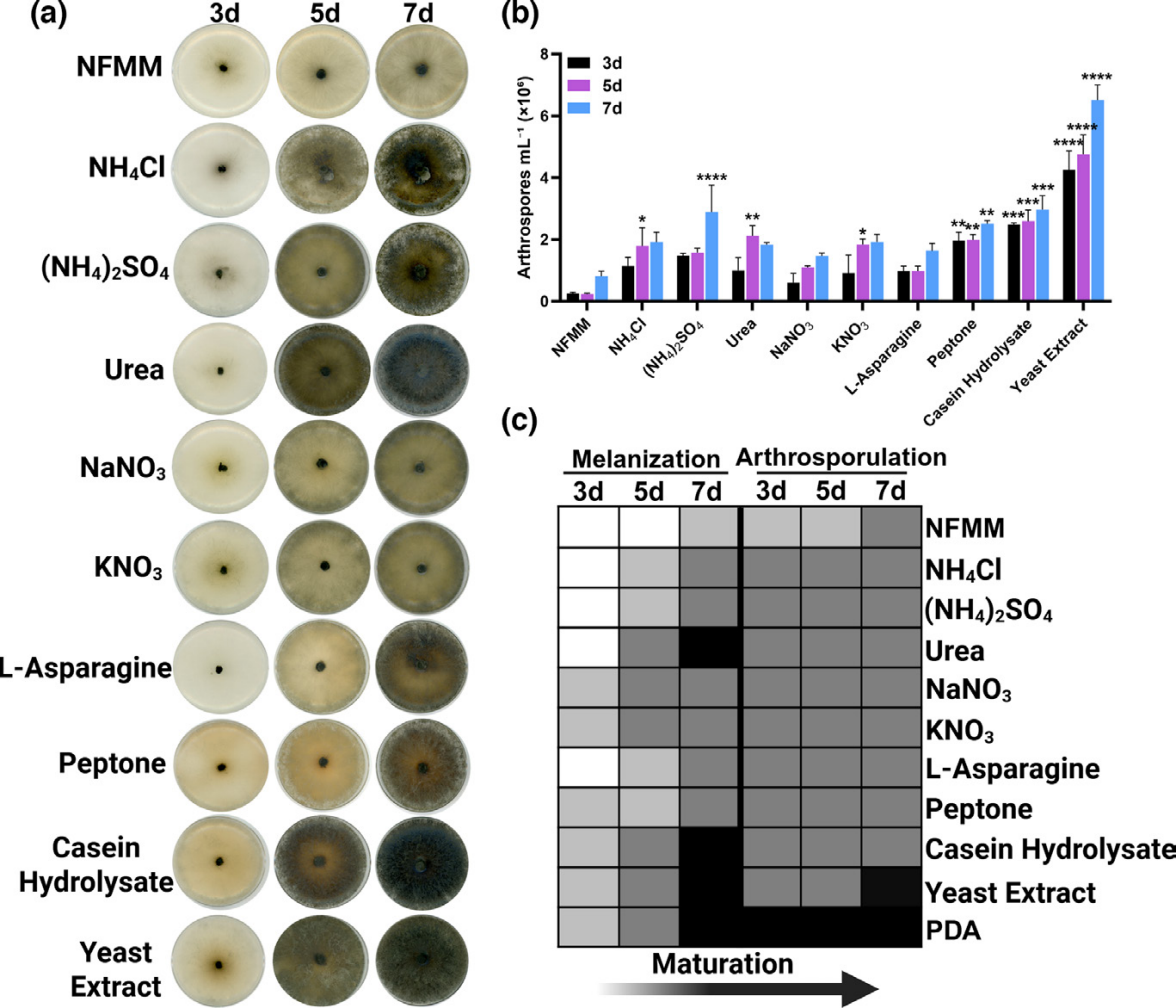
Nitrogen source utilization by *N. dimidiatum*. (a) Representative colony morphology of *N. dimidiatum* grown on nitrogen-free minimal medium (NFMM) supplemented with 0.1% (wt/vol.) individual nitrogen sources at 3, 5 and 7 dpi. **(b)** Quantification of arthrospore production under the indicated nitrogen sources. Statistical significance was determined by two-way ANOVA with Dunnett’s post-hoc test. * indicates *P*<0.05, ** indicates *P*<0.01, *** indicates *P*<0.001 and **** indicates *P*<0.0001. **(c)** Composite heat map summarizing melanization and arthrosporulation scores across nitrogen sources. Darker shading indicates higher scores.

At 3 dpi, colonies grown on peptone, casein hydrolysate and yeast extract exhibited extensive growth, covering most of the plate surface (Fig. 3a). By 5 dpi, robust growth persisted in the casein hydrolysate and yeast extract treatments, accompanied by increased melanization in several other nitrogen sources, particularly ammonium salts, nitrates and urea. At 7 dpi, cultures grown on yeast extract and casein hydrolysate remained the most visually similar, displaying dense mycelial growth and pronounced melanization. Although maturation was evident across multiple nitrogen sources, colonies grown on NH_₄_Cl, (NH_₄_)_₂_SO_₄_, l-Asparagine, urea and peptone exhibited thin peripheral mycelial regions, visible underlying media and localized white patches, consistent with reduced growth (Fig. 3a). Arthrosporulation analysis revealed that yeast extract most strongly supported both growth and conidiation. At 3 dpi, all other nitrogen sources resulted in significantly reduced arthrosporulation compared to yeast extract, with differences becoming more pronounced at 5 and 7 dpi (Fig. 3b). These findings demonstrate that yeast extract was the most favourable nitrogen source, promoting coordinated fungal development and maturation (Fig. 3c).

### *N. dimidiatum* possesses plant cell wall-degrading enzymatic activity

To assess the secretion of plant cell wall-degrading enzymes (PCWDEs) and proteases by *N. dimidiatum*, we performed *in vitro* plate-based substrate degradation assays. Genomic analyses predict that *N. dimidiatum* encodes a diverse repertoire of carbohydrate-active enzymes (CAZymes) and PCWDEs [9]. Combined with our observations of fungal growth on complex carbohydrates and peptide-rich nitrogen sources, we evaluated extracellular cellulase, amylase, pectinase and protease activities at 3 dpi.

**Fig. 4.**
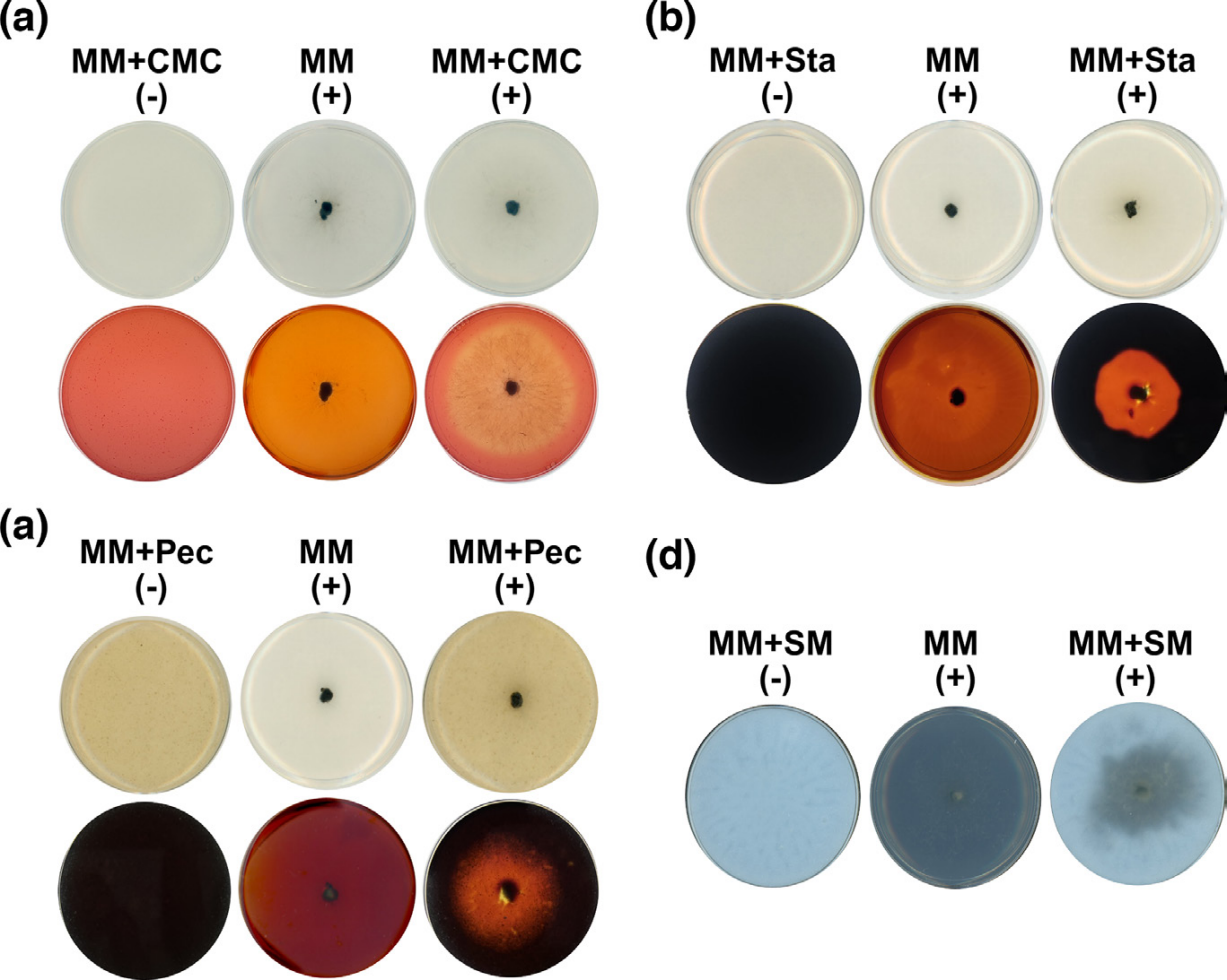
Extracellular enzymatic activity of *N. dimidiatum*. (a) Cellulase activity on modified Czapek-Dox minimal medium (MM) supplemented with 1% (wt/vol.) carboxymethyl cellulose (CMC). **(b)** Amylase activity on MM supplemented with 1% (wt/vol.) starch (Sta). **(c)** Pectinase activity on MM supplemented with 1% (wt/vol.) pectin (Pec). **(d)** Protease activity on MM supplemented with 10% (wt/vol.) skim milk (SM). Uninoculated substrate controls are indicated by (−) and inoculated plates by (+). Images were captured at 3 dpi.

On CMC-amended plates, Congo Red staining followed by NaCl washing revealed a distinct clearance zone surrounding the inoculum (Fig. 4a), consistent with cellulase-mediated CMC degradation [16]. No clearing was observed in control plates, confirming substrate-dependent activity.

Amylase activity was detected on starch-supplemented plates stained with Gram’s iodine, which binds intact starch polymers [17]. A strong clearance halo was observed around the fungal plug (Fig. 4b), indicating starch degradation, in accordance with moderate growth and arthrosporulation on starch-supplemented media in carbon utilization assays (Fig. 2a–c). Similarly, pectin-supplemented plates stained with iodine exhibited localized clearing surrounding the inoculation site, supporting pectinase activity (Fig. 4c).

Protease activity was confirmed on SM agar, where fungal growth produced a clear zone surrounding the inoculum, indicating casein degradation (Fig. 4d). This aligns with the nitrogen utilization assays, where *N. dimidiatum* grew robustly on casein hydrolysate and yeast extract (Fig. 3a–c). Collectively, these results demonstrate that *N. dimidiatum* secretes extracellular enzymes capable of degrading cellulose, starch, pectin and protein substrates under *in vitro* conditions (Fig. 4a–d).

## Discussion

DFC, caused by *N. dimidiatum*, is a major constraint to dragon fruit production. This study provides an initial physiological characterization of carbon and nitrogen utilization preferences in *N. dimidiatum* and confirms secretion of extracellular enzymes associated with plant tissue degradation, offering insight into its physiology and nutrient-acquisition strategies.

Under nutrient-limited conditions, key developmental processes were delayed, particularly melanization and arthrosporulation. These effects were most evident at 5 and 7 dpi, when both traits were markedly reduced relative to PDA-grown cultures. Impaired melanization may have important consequences for fungal fitness, as melanin is associated with environmental stress tolerance and infection-related development in many plant-pathogenic fungi [15,18, 19]. Reduced arthrosporulation may likewise limit inoculum production and disease dissemination. These observations suggest that nutrient availability is an important regulator of developmental traits linked to fitness and persistence in *N. dimidiatum*.

Carbon utilization assays revealed a clear preference for maltose, which supported melanization and arthrosporulation at levels comparable to PDA. Given that PDA is rich in starch-derived carbohydrates, maltose may reflect a carbohydrate environment relevant to host colonization. Maltose, a starch-derived disaccharide, also supported more robust development than glucose alone, suggesting that *N. dimidiatum* may be well adapted to utilize starch-derived sugars [20,21]. Similar maltose preferences have been reported in other plant-pathogenic fungi and ascomycetes, where uptake involves dedicated transporters and intracellular hydrolysis by α-glucosidases [22]. The ability of *N. dimidiatum* to grow on starch-containing media and secrete amylase further supports this interpretation. Because starch is present in pitaya tissues, starch degradation and maltose assimilation may represent biologically relevant strategies during host colonization and disease development, although confirmation will require future molecular and *in planta* studies [23,24].

In contrast, glucose and sucrose supported partial melanization but delayed or reduced arthrosporulation, whereas polyols and lactose resulted in severely impaired growth. These differences likely reflect limitations in carbohydrate uptake or metabolic entry into glycolysis, as certain substrates do not readily feed into central carbon pathways [25,26]. Altered carbon metabolism may influence cyclic AMP-protein kinase A signalling, a pathway known to regulate melanin biosynthesis and fungal development [27], providing a plausible explanation for these phenotypes, although additional studies are needed to define the underlying mechanisms.

Nitrogen utilization assays demonstrated a clear preference for complex, peptide-rich sources, with yeast extract supporting the most robust growth, melanization and arthrosporulation. Because yeast extract contains peptides, amino acids, vitamins and trace elements, its efficient utilization suggests that readily assimilable and nutritionally complex nitrogen sources strongly influence fungal development [9,28]. Melanization observed on ammonium-based sources further indicates that *N. dimidiatum* can utilize inorganic nitrogen forms, although delayed arthrosporulation and localized white patches under these conditions suggest that additional nutritional components may be required for optimal development [29]. The preference for peptide-rich substrates also aligns with the extracellular protease activity detected in this study, supporting a possible role for host protein degradation in nitrogen acquisition during infection [30].

Consistent with these metabolic preferences, *N. dimidiatum* secreted multiple extracellular enzymes, including cellulases, pectinases, amylases and proteases. These findings corroborate genomic predictions of CAZyme and PCWDE repertoires [9,21] and support their likely contribution to host colonization. Because cellulose, starch and pectin are major structural components of pitaya tissues, their degradation may facilitate nutrient acquisition and tissue colonization during infection. Similar enzymatic strategies are widely reported in fungal plant pathogens and have been associated with host penetration, colonization and sporulation [31,35]. Extracellular protease activity may further support nitrogen acquisition and additional infection-related processes [33,37].

Collectively, these findings show that *N. dimidiatum* exhibits distinct carbon and nitrogen utilization preferences and secretes extracellular enzymes capable of degrading plant-associated carbohydrates and proteins. Nutrient limitation also impaired melanization and arthrosporulation, reinforcing the relationship between metabolic capacity and fungal fitness. Future studies should examine carbon catabolite repression, host-derived nitrogen utilization and the role of individual degradative enzymes during infection using molecular and *in planta* approaches to improve management of DFC.

## Abbreviations

CAZymes: carbohydrate-active enzymes
CFMM: carbon-free minimal medium
CFW: calcofluor white
CMC: carboxymethylcellulose
DFC: dragon fruit canker
dpi: days post-inoculation
MM: minimal medium
NFMM: nitrogen-free minimal medium
PCWDE: plant cell wall-degrading enzyme
PDA: potato dextrose agar
SM: skim milk
